# Molecular phylogenetic analysis of nuclear genes suggests a Cenozoic over-water dispersal origin for the Cuban solenodon

**DOI:** 10.1038/srep31173

**Published:** 2016-08-08

**Authors:** Jun J. Sato, Satoshi D. Ohdachi, Lazaro M. Echenique-Diaz, Rafael Borroto-Páez, Gerardo Begué-Quiala, Jorge L. Delgado-Labañino, Jorgelino Gámez-Díez, José Alvarez-Lemus, Son Truong Nguyen, Nobuyuki Yamaguchi, Masaki Kita

**Affiliations:** 1 Laboratory of Animal Cell Technology, Faculty of Life Science and Technology, Fukuyama University, Higashimura-cho, Aza, Sanzo, 985, Fukuyama 729-0292, Japan; 2 Institute of Low Temperature Science, Hokkaido University, Kita-19 Nishi-8, Kita-ku, Sapporo 060-0819, Japan; 3 Environmental Education Center, Miyagi University of Education, Aramaki Aza-Aoba, Aoba-ku, Sendai 980-0845, Japan; 4 Sociedad Cubana de Zoología, CP 11900, Boyeros, La Habana, Cuba; 5 Unidad Presupuestada Parque Nacional Alejandro de Humboldt (CITMA), Calle Abogado 14 e/ 12 y 13 Norte, Guantanamo 95200, Cuba; 6 Estación Ecológica La Melba, Unidad Presupuestada Parque Nacional Alejandro de Humboldt, CITMA-Guantánamo, Cuba; 7 Centro de Inspección y Control Ambiental (CICA), Ministerio de Ciencia, Tecnología y Medio Ambiente (CITMA), Cuba; 8 Institute of Ecology and Biological Resources, Vietnam Academy of Science and Technology, 18 Hoang Quoc Viet Street, Cau Giay, Hanoi, Vietnam; 9 Department of Biological and Environmental Sciences, College of Arts and Sciences, Qatar University, PO Box 2713 Doha, Qatar; 10 Graduate School of Pure and Applied Sciences and Tsukuba Research Center for Interdisciplinary Materials Science (TIMS), University of Tsukuba 1-1-1 Tennodai, Tsukuba 305-8571, Japan

## Abstract

The Cuban solenodon (*Solenodon cubanus*) is one of the most enigmatic mammals and is an extremely rare species with a distribution limited to a small part of the island of Cuba. Despite its rarity, in 2012 seven individuals of *S. cubanus* were captured and sampled successfully for DNA analysis, providing new insights into the evolutionary origin of this species and into the origins of the Caribbean fauna, which remain controversial. We conducted molecular phylogenetic analyses of five nuclear genes (*Apob*, *Atp7a*, *Bdnf*, *Brca1* and *Rag1*; total, 4,602 bp) from 35 species of the mammalian order Eulipotyphla. Based on Bayesian relaxed molecular clock analyses, the family Solenodontidae diverged from other eulipotyphlan in the Paleocene, after the bolide impact on the Yucatan Peninsula, and *S. cubanus* diverged from the Hispaniolan solenodon (*S. paradoxus*) in the Early Pliocene. The strikingly recent divergence time estimates suggest that *S. cubanus* and its ancestral lineage originated via over-water dispersal rather than vicariance events, as had previously been hypothesised.

As a natural laboratory, the Caribbean islands have garnered considerable attention worldwide because of their unique biogeographic features that have contributed to our understanding of organismal evolution^1^,^2^. In particular, the species assembly rule of Caribbean biota has been a subject of intensive research and discussion. Since the early 20^th^ century, there have been long-standing debates between vicariance and dispersal mechanisms of origin for the Caribbean species assembly^2^,^3^,^4^,^5^,^6^,^7^,^8^,^9^,^10^. The most heated argument has been whether land bridges between the Caribbean islands and nearby continents in the Cenozoic Era contributed to the colonisation of organisms onto the islands. Previous geological studies proposed the GAARlandia hypothesis, in which a land bridge was formed between the Greater Antilles (Cuba, Hispaniola, Jamaica and Puerto Rico) and South America via the Aves Ridge (AR) around 33–35 Ma (mega annum)^7^,^11^. Supporting evidence for GAARlandia has been obtained for various species^9^,^12^,^13^,^14^,^15^,^16^; however, these studies did not provide exclusive support for this hypothesis, either because dispersal can also explain the results^9^,^12^,^13^,^15^, or the data do not precisely coincide with the time scale of GAARlandia (33–35 Ma)^14^,^16^. However, most empirical studies on vertebrate species have supported the over-water dispersal hypothesis for the origin of West Indian biota^4^,^6^,^10^,^17^,^18^, rather than overland dispersal and subsequent vicariance in GAARlandia. In addition, a vicariant event unrelated to the GAARlandia hypothesis has been suggested for freshwater cichlid fish species between the Greater Antilles and the Yucatan Peninsula in Central America^19^. Therefore, the colonisation mechanism of the Caribbean biota remains elusive, and a generalisation of the species establishment process is unresolved.

The Cuban solenodon or almiquí (*Solenodon cubanus*) is a large insectivore (Fig. 1) and an endangered species isolated on the island of Cuba^20^. It is listed as Endangered B1ab(iii,v) on the IUCN Red List, indicating that its distribution is severely fragmented in several limited locations and the population continues to decline^21^,^22^. Moreover, the capture record of live solenodons is extremely poor, making this species one of the most enigmatic mammals worldwide. Therefore, basic biological data are required for their conservation. Together with the Hispaniolan solenodon (*S. paradoxus*), these two extant species constitute the family Solenodontidae in the order Eulipotyphla^23^. Because the fossil record of Solenodontidae, especially the genus *Solenodon*^24^, is limited, the evolution and systematic relationships within Eulipotyphla cannot be accurately investigated without access to living solenodon individuals. Clarifying the establishment process of this species in Cuba could contribute to resolving the Caribbean faunal assembly rule.

There have been only a few investigations into the origin of the Cuban solenodon. The divergence time between the Cuban and Hispaniolan solenodons was estimated to be 25 Ma (95% credibility interval [CI] = 16–38 Ma) based on a molecular chronological analysis of mitochondrial DNA (mtDNA) using old museum specimens^25^. Roca *et al.*^25^ indicated that there is consistency between the molecular estimate and geological evidence that the separation between Cuba and Hispaniola occurred at 25–27 Ma^7^ and suggested that the two solenodon species could have originated via a vicariance event. However, there are two problems with their conclusion. First, the geological evidence has been updated and it was inferred that the separation between these two islands occurred ~16 Ma^11^, which does not support their molecular estimate of 25 Ma, although the 95% CI marginally covers 16 Ma^25^. Second, only mtDNA was examined in their study on the Cuban solenodon, despite using multiple nuclear DNA (nucDNA) genes for other eulipotyphlan species. Dating old divergences with mtDNA is known to be problematic because of the rapid evolutionary rate of mtDNA^26^. It is well known that genetic analyses of old museum samples are difficult due to the degraded state of the DNA samples, especially for nucDNA genes^27^. Under such conditions, only mtDNA with large copy numbers have been examined. The more slowly evolving nucDNA would provide better insight into the time scale of solenodon evolution; however, to analyse nucDNA, fresh tissue samples are necessary. Recently, we successfully obtained fresh tissue samples of the rare Cuban solenodon (see Methods), which allowed for nucDNA sequence analyses.

For the phylogenetic and chronological analyses in this study, we used five exon regions of slowly evolving nucDNA genes (*Apob*, apolipoprotein B; *Atp7a*, ATPase, Cu++ transporting, alpha polypeptide; *Bdnf*, brain-derived neurotrophic factor; *Brca1*, breast cancer 1, early onset; *Rag1*, recombination activating gene 1), all of which have been used in previous molecular systematic studies of mammals^25^,^28^,^29^,^30^,^31^. Our analysis of the phylogenetic relationships and divergence times using a molecular supermatrix composed of the five nuclear gene exon sequences provided evidence supporting over-water dispersal, rather than vicariance, for the origin of the family Solenodontidae in the Paleocene and the Cuban solenodon in the Early Pliocene.

## Results and Discussion

### Origin of Solenodontidae

**Figure 1:**
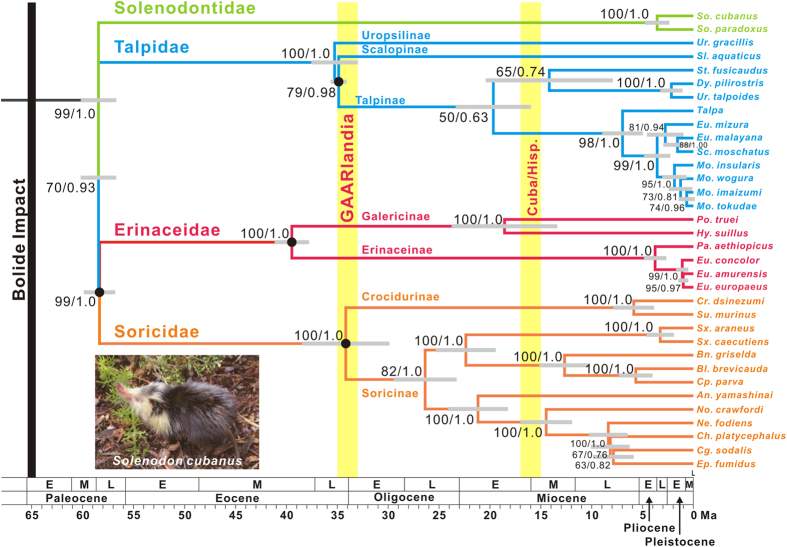
Bayesian chronogram of the order Eulipotyphla. The divergence times were estimated from the BEAST analyses of five nuclear genes (*Apob*, *Atp7a*, *Bdnf*, *Brca1* and *Rag1*; total, 4,602 bp). The maximum likelihood (ML) and Bayesian inference (BI) analyses both supported the same topology, as shown. The colours of each branch show four families in this order (green, Solenodontidae; blue, Talpidae, red, Erinaceidae; orange, Soricidae). Numbers near each node show bootstrap supports (left) and posterior probabilities (right) from the ML and BI analyses, respectively. The geological time scale for the Cenozoic era (65 Ma–present) is shown at the bottom and several geological events are marked on the chronogram by black or yellow thick vertical lines (Bolide Impact at 65 Ma, GAARlandia at 33–35 Ma, and geological separation between Cuba and Hispaniola [Cuba vs Hisp.] at ~16 Ma). The letters E, M and L in the time scale indicate Early, Middle and Late, respectively. The 95% credibility intervals for each divergence time estimate are represented by the grey horizontal bars. The nodes constrained for dating are represented by the black circles on each node. For details of the calibration settings, see the Methods section. The image of *Solenodon cubanus* was photographed by one of the authors (L.M.E).

The supermatrix phylogenetic analyses of five exon regions of nucDNA genes (total, 4,602 bp) demonstrated that Solenodontidae diverged first among the four families of Eulipotyphla (Solenodontidae, Erinaceidae, Soricidae and Talpidae^23^; Fig. 1 and Supplementary Fig. S1; bootstrap proportion = 70, posterior probability = 0.93), followed by Talpidae, with Erinaceidae and Soricidae forming a clade. This is congruent with one previous study^25^, but contradicts another supporting a phylogenetic affinity between Soricidae and Talpidae using 26 nuclear genes (total, 35,603 bp)^31^. Despite the difference in some of the inter-familial relationships, the basal position of Solenodontidae was robust for the examined gene sequences among these studies, including ours. Conversely, previous studies might have encountered the long-branch attraction (LBA)^32^ problem because only one or two representative species of each family separated by long branches were examined. For the phylogenetic and chronological analyses of this study, we sampled all four families in Eulipotyphla and all of the subfamilies in each family, where available (Erinaceinae and Galericinae in Erinaceidae, Crocidurinae and Soricinae in Soricidae, and Scalopinae, Talpinae, and Uropsilinae in Talpidae; Supplementary Table S1). Using this sampling procedure, we conducted analyses using all of the major lineages in Eulipotyphla at the subfamilial level, making the phylogenetic analyses less vulnerable to the LBA problem for inter-familial relationships. Therefore, our analyses using all subfamilial lineages largely strengthened the hypothesis of the basal branching of Solenodontidae and the close affinity of Erinaceidae and Soricidae. The phylogenetic inconsistency between this study and that by Meredith *et al.*^31^ and the very short branch lengths among the four major families in the chronogram (Fig. 1) suggest that rapid cladogenesis within a short evolutionary period was involved in these divergences; thus, further data are required for complete resolution of the inter-familial phylogenetic relationships.

The vicariance and dispersal hypotheses have been intensively debated as the origin of Solenodontidae. In recent molecular phylogenetic studies, the emergence of the ancestral lineage of Solenodontidae was inferred to have occurred in the late Cretaceous period (76 Ma [95% CI = 72–81 Ma] in Roca *et al.*^25^; 77.3 Ma [95% CI = 70.7–85.8 Ma] in Meredith *et al.*^31^), which is chronologically consistent with the previous supposition that vicariance and subsequent restriction to the proto-Antilles, located between North and South America (near current Central America), in the Late Cretaceous period^6^,^33^ generated the ancestral lineage of Solenodontidae^10^. This is not a new hypothesis, since Rosen^33^ depicted the hypothetical relative movements of the proto-Antilles northeastward from the region of the present Central America. However, palaeogeographical evidence is insufficient to support the proto-Antillean vicariance hypothesis^6^. Moreover, it seems implausible that ancestral solenodon populations could have survived the devastating impacts of the gigantic bolide on the Yucatan Peninsula (Chicxulub) at 65 Ma^3^,^4^,^6^,^11^,^17^, which was also a concern of Roca *et al.*^25^ in their discussion of the late Cretaceous divergence of Solenodontidae.

In this study, we tested the hypothesis of a Late Cretaceous origin of Solenodontidae with multiple fossil calibrations within Eulipotyphla^34^, which was accomplished using our extensive sampling strategy. Using only eulipotyphlan fossil calibrations, we obtained an estimate of 58.6 Ma (95% CI = 57.3–60.8 Ma) for the age of the most recent common ancestor (TMRCA) of Eulipotyphla (Fig. 1 and Supplementary Table S2; strategy [1]), which is later than the age of the bolide impact (65 Ma; Fig. 1). This estimate is also incongruent with the GAARlandia land bridge in the Eocene-Oligocene boundary period (33–35 Ma; Fig. 1). Therefore, it supports the possibility that early Cenozoic dispersal after the bolide impact, not Late Cretaceous proto-Antillean vicariance, produced the ancestral lineage of Solenodontidae as previously suggested^4^, although incomplete fossil records could promote underestimation of the divergence times. Supporting this, the North American origin of the over-water dispersal was suggested based on fossil evidence^35^. Such an over-water dispersal origin of extinct North American soricomorph relatives has been considered a possible explanation for the current relictual distribution of Solenodontidae^7^.

Furthermore, Cenozoic intra-ordinal divergence after the Cretaceous-Cenozoic boundary (65 Ma) is a common trend in mammalian diversification^31^. Interestingly, Phillips^36^ re-analysed the dataset of Meredith *et al.*^31^, taking into consideration the calibration and evolutionary rate errors among the major mammalian lineages, and produced a divergence time estimate for the TMRCA of Eulipotyphla, which is consistent with this study (59.1 Ma). Geologists have stated that the Greater Antilles was submerged until the Middle Eocene (ca 45 Ma)^7^,^37^, and early mammalian fossils in the region date back to the Oligocene^22^, which is incongruent with the hypothesis of the proto-Antillean vicariance in the late Cretaceous period. However, it is also inconsistent with our estimate for the origin of ancestral Solenodontidae (58.6 Ma [95% CI = 57.3–60.8 Ma]). Currently, the idea that the ancestor of Solenodontidae originated from another location by dispersal no earlier than 58.6 Ma (after the emergence of the Greater Antilles) and “relictualised” might be the best hypothesis explaining the absence of fossil traces of the major continental groups in these islands that would otherwise have dispersed through the land bridge^7^. Nevertheless, further independent molecular and palaeontological evidence or geological updates are necessary for more precise dating of the origin of Solenodontidae. Old divergences estimated in two previous studies may have been due to the use of completely or mostly non-eulipotyphlan fossils as calibration points in the estimation of divergence times among eulipotyphlan lineages^25^,^31^, as discussed in Phillips^36^. A recently published study also indicated a Late Cretaceous origin for Solenodontidae (78.7 Ma [95% CI = 62.1–98.6 Ma])^38^. However, this study used the same fossil constraints as those of Meredith *et al.*^31^. Therefore, the time estimate of the Late Cretaceous age might be another example of overestimation.

### Origin of the Cuban solenodon

The Cuban solenodon formed a clade with the Hispaniolan solenodon (Fig. 1). This relationship was highly supported by the bootstrap value (100%) and posterior probability (1.0) in the maximum-likelihood (ML) and Bayesian-inference (BI) analyses, respectively (Fig. 1 and Supplementary Fig. S1). Although we used various calibration standards (see Methods), the divergence times between the Cuban and Hispaniolan solenodons estimated by the Bayesian relaxed molecular clock analysis were robust to the difference in calibrations, demonstrating that the two solenodons diverged in the Early Pliocene (3.7–4.8 Ma [95% CI = 2.6–6.4 Ma]; Supplementary Table S2). This estimate is incongruent with the previous estimate (25 Ma [95% CI = 16–38 Ma])^25^, which was used to suggest that the geological separation between Cuba and Hispaniola (25–27 Ma)^7^ would have led to the vicariance event between these species. Despite using 76 Ma for the basal divergence of the eulipotyphlan clade to compare the time scales between previous studies and our results (strategies [2] and [3]; see Methods), the basal divergence within Solenodontidae (*S. cubanus* versus *S. paradoxus*) was still estimated to have occurred in the Early Pliocene (4.6–4.8 Ma [95% CI = 3.1–6.4 Ma]; Supplementary Table S2), strikingly different from the previous estimate (25 Ma [95% CI = 16–38 Ma])^25^.

It could be argued that the use of mtDNA caused overestimations in the divergence times of Roca *et al.*^25^. Since mtDNA evolves much more rapidly than nucDNA, the saturation of substitutions becomes problematic when using mtDNA to estimate divergence times among distantly related species^26^. Saturation leads to underestimation of substitutions, resulting in overestimation of divergence times among younger lineages than the calibration points^39^. This could be the case for the previous mtDNA dating analysis^25^, in which the use of mtDNA was unavoidable because of the old museum samples of *S. cubanus* used for the genetic analyses. Therefore, the younger estimate obtained using the more slowly evolving nucDNA in this study is considered to be a reasonable time estimate and we conclude that the Cuban solenodon originated in the Early Pliocene. It should be noted that although there is a two-fold difference in the branch lengths between the Cuban and Hispaniolan solenodon lineages in the ML tree topology (Supplementary Fig. S1), it does not pose a major problem because of the robustness of the time estimate obtained using our drastically different calibration strategy. The difference between the Cuban and Hispaniolan solenodons is only 1.2% (56 of 4602 bp). This small difference might have stochastically affected the branch length variation.

The Early Pliocene origin of the Cuban solenodon supports over-water dispersal from Hispaniola as the mechanism for its origin on the island of Cuba for several reasons. First, there have been no geological reports that imply land connections between Cuba and Hispaniola later than the Early to Middle Miocene (16 Ma)^11^. Second, since the unidirectional ocean current around the Caribbean Sea flows northwestward from South America^4^, it is reasonable to assume that the Cuban lineage dispersed from Hispaniola, not the opposite. However, from a geological perspective, the present ocean surface current patterns have been suggested to be a feature of the last 4 million years^7^. Based on this argument, the over-water dispersal hypothesis has been criticised because the divergence was mostly dated to be >4 Ma^4^,^7^. Conversely, the estimate obtained in this study (3.7–4.8 Ma) is not contradicted by the geological evidence that the current ocean circulation was initiated in the Pliocene period^7^. Third, a number of studies have supported the over-water dispersal hypothesis as the origin of the West Indian biota, especially non-volant mammals, reptiles and amphibians^4^,^6^,^17^,^18^. Traditional examples for the over-water dispersal hypothesis have been provided by immunological-distance analysis^5^,^6^,^17^, whereby most of the origins of West Indian vertebrates are not concentrated in a particular period, implying little or no relation to specific geological land bridges. A recent empirical study using a museomics approach with next-generation sequencing technology supported the over-water dispersal of hutias (capromyine rodents with body sizes similar to solenodons) from South America^40^. In that study, Hispaniola was suggested as a source for the later diversification of hutias in the Greater Antilles and Bahamas, which is in agreement with the dispersal direction inferred for solenodons in this study. Additional supportive evidence for the over-water dispersal origin from other islands, continents, and even from Caribbean islands has accumulated recently for a variety of taxonomic groups in the West Indies, including *Poitea* and *Erithalia* plants^41^, pupfishes^42^, eleutherodactyline frogs^43^, *Anolis* lizards^44^, alsophiine racer snakes^45^, extinct oryzomyine rodents^46^, short-faced bats^47^ and platyrrhine primates^48^. Therefore, our case study of *Solenodon* provides additional support for the dispersal origin of Caribbean vertebrates. All available evidence in recent molecular phylogenetic studies has shown that smaller invertebrates and a few vertebrates support the hypotheses of GAARlandia (*Selenops* spiders^12^, butterflies^15^, orb-weaving spider^16^, cichlid fishes^14^, toads^13^) and other geological vicariance events (crickets^49^), while most vertebrates with relatively larger body sizes support the over-water dispersal as a major role in their origins, suggesting that the biological properties of species could have affected the over-water dispersal processes of West Indian biota. This is consistent with a previous study that found that more distant island voles are larger in size than mainland or less distant island voles^50^. This does not mean that smaller species were mostly affected by geological vicariance events, but rather that there may have been few survivors among smaller species in the high-risk passage of over-water dispersal.

## Methods

### Sample collection

The field survey was conducted in Alejandro de Humboldt National Park in the Republic of Cuba from March to April 2012, resulting in the capture of seven live Cuban solenodon individuals (Permit No. 03/2012 from the Cuban Environment Agency to collect DNA samples and export them to Japan, document available upon request). After collecting the data, all individuals were carefully released into their habitat. All methods followed the approved guidelines provided by the Mammal Society of Japan. Including these newly captured seven Cuban solenodons, the nucleotide sequences of five exon regions of nucDNA genes, *Apob*, *Atp7a*, *Bdnf*, *Brca1* and *Rag1*, were examined for 35 species in the order Eulipotyphla (Supplementary Table S1). For the phylogenetic and chronological analyses, we obtained novel sequences from our experiments or downloaded previously determined sequences from the DDBJ/EMBL/GenBank international DNA databases (Supplementary Table S1). Three species in the order Carnivora (the Japanese weasel, *Mustela itatsi*, the ermine or stoat, *Mustela erminea*, and the small Asian mongoose, *Herpestes auropunctatus*), one species in Scandentia (the northern tree shrew, *Tupaia belangeri*) and one species in Rodentia (the house mouse, *Mus musculus*) were used as outgroups in the phylogenetic analyses. Eulipotyphla and Carnivora were classified into the superorder Laurasiatheria, while the other Scandentia and Rodentia were classified into Euarchontoglires, the sister clade of Laurasiatheria^31^. These outgroups span taxonomically diverse groups and are expected to work well to fix the eulipotyphlan root.

### Experimental techniques

Total genomic DNA was extracted from a piece of ear tissue using a standard phenol–chloroform method. All PCR reactions were performed with the KOD-Plus-NEO DNA Polymerase Kit (Toyobo, Osaka, Japan) in an automated thermal cycler (Life Touch thermal cycler, Bioer Technology, Hangzhou, China) using the following conditions: a 3-min denaturation period at 94 °C, 30 cycles of denaturation at 94 °C for 30 s, annealing at 50 °C for 30 s, extension at 68 °C for 90 s and a 10-min extension period at 68 °C. The PCR reaction mixture consisted of KOD-Plus-NEO buffer, 1.5 mM MgSO_4_, 0.2 mM of each dNTP, 0.3 μM of forward and reverse primers for each gene (Supplementary Table S3), 1.0 U of KOD-Plus-Neo DNA polymerase and 0.1–0.2 μg of template total genomic DNA to a total volume of 50 μL. Nested PCR reactions were performed for *Apob*, *Brca1* and *Rag1* using each first and second PCR primer pair in two PCR steps, while non-nested reactions for *Atp7a* and *Bdnf* used only one pair of primers in one PCR step (Supplementary Table S3). In the second PCR, a 1-μL aliquot of each first PCR product was used as a template. The PCR conditions were the same as the first PCR. We sequenced the ca. 500–700 bp PCR products using the Big Dye Terminator Cycle Sequencing Kit ver. 3.1 (Thermo Fisher Scientific, Tokyo, Japan), followed by the ABI3130 automated sequencer (Applied Biosystems, Tokyo, Japan). All experimental protocols were approved by the Animal Care and Use Committee of Fukuyama University.

### Construction of the molecular supermatrix

Multiple alignments of the determined sequences were conducted for each locus through the program MUSCLE implemented in MEGA ver. 6^51^. Then, we combined alignments of each locus into a single data matrix (supermatrix) containing 40 species and five nuclear gene sequences (*Apob*, 1,181 bp; *Atp7a*, 673 bp; *Bdnf*, 573 bp; *Brca1*, 1,071 bp; and *Rag1*, 1,104 bp; total, 4,602 bp). The supermatrix was composed of 230,100 taxon-by-character cells, where the number of missing cells was 73,351 (31.9%). Missing data were not relevant to the phylogenetic resolution, as the most important point in phylogenetic design is the informativeness of the collected data^52^,^53^. Even data matrices with >60% missing cells can provide a well-resolved phylogeny if there are informative sequences in the data matrix^52^.

### Phylogenetic analyses

We adopted two probabilistic phylogenetic inference approaches, ML and BI, using the constructed molecular supermatrix. Before conducting these phylogenetic analyses, we inferred the best-fit partitioning scheme and substitution models using PartitionFinder ver. 1.1.1^54^. Partitions were pre-defined by genes and codons (15 partitions; five genes and three codon positions), and the best partitioning scheme was explored using the greedy search and determined by the Bayesian information criterion. The best partitioning scheme supported two subsets of partitions, where SYM+G was selected for the first and second codon positions of the *Bdnf* and *Rag1* genes, while GTR + I + Γ was used for the other 11 partitions. The ML phylogenetic analysis was conducted in GARLI ver. 0.96^55^ using these combined substitution models. The ML tree was inferred through heuristic searches consisting of five independent runs of the genetic algorithm, each performing 2 × 10^4^ generations of mutation–selection–reproduction cycles with the starting tree generated from the ML stepwise–addition–sequence option. We followed the default option for the other parameters. Nodal support was evaluated using nonparametric bootstrap analyses (100 replicates).

The BI phylogenetic analysis was conducted in MrBayes ver. 3.1.2^56^. The best-fit substitution models were inferred as above. The model parameters were estimated during the analysis. Gene partitions were unlinked. The substitution rates were allowed to vary across partitions with the *prset ratepr* = *variable* option. We conducted two independent runs of Metropolis-coupled Markov-chain Monte Carlo (MCMC). Each run consisted of four Markov chains, one cold and three incrementally heated, starting from a random tree. The chains were run for 1 × 10^6^ generations and sampled every 10^2^ generations. The first 2.5 × 10^3^ sampled trees (25%) were discarded because they were likely to have been sampled during the burn-in period where the posterior probability did not reach the stationary distribution. Tracer ver. 1.6^57^ was used to check that the log likelihood (ln *L*) scores had converged on a stationary distribution. The average standard deviation of split frequencies was 0.001871, indicating convergence of Metropolis-coupled MCMC (<0.01 recommended in the instructions of MrBayes ver. 3.1.2).

### Chronological analyses and fossil calibration points

The chronological analyses were conducted with the Bayesian uncorrelated lognormal relaxed-clock model implemented in BEAST ver. 1.6.2^57^,^58^. The input file was generated in BEAUti ver. 1.6.2^57^. The best-fit substitution models for each gene partition were the same as the BI phylogenetic analysis. The Yule process of speciation was set as a tree prior. Multiple fossil calibration points were set^59^,^60^. Our sampling of diverse familial and subfamilial lineages within Eulipotyphla permitted the use of multiple eulipotyphlan fossils for some families and subfamilies^34^. Roca *et al.*^25^ examined *S. cubanus* using only non-eulipotyphlan fossils as calibration points to estimate divergence times among eulipotyphlans, implying the possibility that the substitution rate in the eulipotyphlan lineages may not have been estimated accurately. This is because evolutionary rates differ among mammalian orders^36^, and the lineage-specific rate variations can strongly influence estimated divergence times^31^,^36^. Therefore, using non-eulipotyphlan fossils in the estimation of divergence times among eulipotyphlans is not preferable. In this study, according to the multiple fossil information^34^, we set 57–59, 37–49, 25–33 and 34–37 Ma as the TMRCA of the Erinaceidae-Soricidae, Erinaceidae, Soricidae and Talpinae-Scalopinae clades, respectively, as calibration points for each lineage, which are phylogenetically closer to the focal lineages than previously examined and reduce the effect of evolutionary rate variation. The minimum ages of the fossil time range (57, 37, 25 and 34, respectively) were set to the zero offset value of the lognormal distribution, assuming the minimum bound of each clade. The mean value was set to 1, 6, 4 and 1.5, respectively, where the mean took the mid-value of each fossil range (e.g., 6 = 43 [mid-value of 37 and 49] - 37). The standard deviations of the lognormal distributions were adjusted so that the 95% quantile took each maximum age (59, 49, 33 and 37, respectively). Hereafter, this calibration is ‘Assumption 1’. In addition, we used 76 Ma as the TMRCA of the Eulipotyphla clade, as estimated previously^25^. In addition, 76 Ma was used as the mean for the normal prior distribution, setting the standard deviation so that the 95% credible interval ranged from 75 to 77 Ma. Hereafter, this calibration is ‘Assumption 2’. In this study, we estimated the divergence times with three calibration strategies, [1] Assumption 1 only, [2] both Assumptions 1 and 2, and [3] Assumption 2 only. Strategy [1] tested the previous estimate of 76 Ma as the basal divergence time of Eulipotyphla without using 76 Ma as a calibration point. Strategy [3] assumed a similar time scale as Roca *et al.*^25^ to test the influence of the difference between mtDNA^25^ and nucDNA (this study) on the divergence times. Finally, using strategy [2], we used all available calibration standards. We conducted three independent MCMC runs of 1 × 10^7^ generations in BEAST, where each parameter was sampled every 10^3^ generations. The BEAST log file was assessed using Tracer 1.6 to confirm the parameter convergence to the stationary distribution and check the efficient sample size (ESS) for each parameter. The initial 25% of samples from each run were excluded as those from the burn-in period. Then, the post-burn-in samples from the three runs were combined. Most ESS values for the TMRCA parameters exceeded 200 (almost all >150), with a few exceptions of ESS <100 (Supplementary Table S2).

## Additional Information

**How to cite this article**: Sato, J. J. *et al.* Molecular phylogenetic analysis of nuclear genes suggests a Cenozoic over-water dispersal origin for the Cuban solenodon. *Sci. Rep.* **6**, 31173; doi: 10.1038/srep31173 (2016).

## Supplementary Material

Supplementary Information
