# Supplementary material for: Molecular phylogenetic analysis of nuclear genes suggests a Cenozoic over-water dispersal origin for the Cuban solenodon
Source: Sci Rep. 2016 Aug 8;6:31173. doi: 10.1038/srep31173 (PMC4976362; doi:10.1038/srep31173)
Supplement: Supplementary Information [file srep31173-s1.pdf]

**Supplementary Information**

# Molecular phylogenetic analysis of nuclear genes suggests a Cenozoic over-water dispersal origin for the Cuban solenodon

Jun J. Sato, Satoshi D. Ohdachi, Lazaro M. Echenique-Diaz, Rafael Borroto-Páez, Gerardo Begué-Quiala, Jorge L. Delgado-Labañino, Jorgelino Gámez-Díez, José Alvarez-Lemus, Nguyen Truong Son, Nobuyuki Yamaguchi, and Masaki Kita

Supplementary Figure S1

Maximum likelihood tree of the order Eulipotyphla. The tree was estimated from the GARLI analysis and the negative log-likelihood score was -31415.497726. Intraspecific variations are shown where relevant. The colours of each branch show four families in this order (green, Solenodontidae; blue, Talpidae, red, Erinaceidae; orange, Soricidae). Numbers near each node show bootstrap supports (before slash) and posterior probabilities (after slash) from the ML and BI analyses, respectively. Numbers in bold near each node are node-numbers, which correspond to those in Supplementary Table S2.

## Supplementary Figure S1

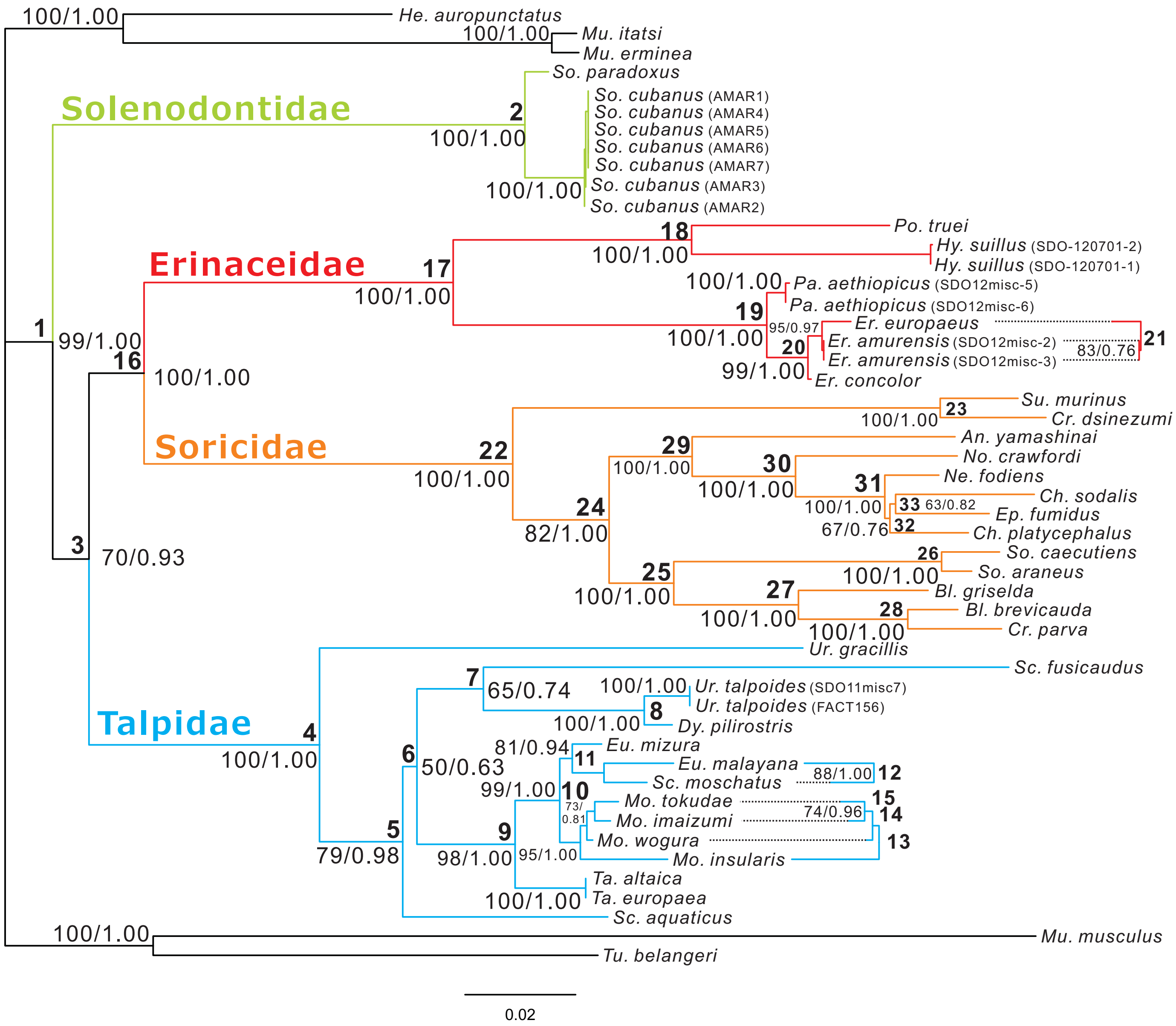

Supplementary Table S1. Species and DNA sequences examined in this study

| Taxon[a] | Voucher number and locality[b] | Accession numbers in the DNA databases | | | | |
|---|---|---|---|---|---|---|
| | | *Apob* | *Atp7a* | *Bdnf* | *Brca1* | *Rag1* |
| **Solenodontidae** | | | | | | |
| *Solenodon cubanus* , Cuban solenodon or Almiqui | AMAR1: AHNP[c], Cuba | LC124826[d] | LC124853[d] | LC124882[d] | LC124915[d] | LC124949[d] |
| *Solenodon cubanus* , Cuban solenodon or Almiqui | AMAR2: AHNP[c], Cuba | LC124827[d] | LC124854[d] | LC124883[d] | LC124916[d] | LC124950[d] |
| *Solenodon cubanus* , Cuban solenodon or Almiqui | AMAR3: AHNP[c], Cuba | LC124828[d] | LC124855[d] | LC124884[d] | LC124917[d] | LC124951[d] |
| *Solenodon cubanus* , Cuban solenodon or Almiqui | AMAR4: AHNP[c], Cuba | LC124829[d] | LC124856[d] | LC124885[d] | LC124918[d] | LC124952[d] |
| *Solenodon cubanus* , Cuban solenodon or Almiqui | AMAR5: AHNP[c], Cuba | LC124830[d] | LC124857[d] | LC124886[d] | LC124919[d] | LC124953[d] |
| *Solenodon cubanus* , Cuban solenodon or Almiqui | AMAR6: AHNP[c], Cuba | LC124831[d] | LC124858[d] | LC124887[d] | LC124920[d] | LC124954[d] |
| *Solenodon cubanus* , Cuban solenodon or Almiqui | AMAR7: AHNP[c], Cuba | LC124832[d] | LC124859[d] | LC124888[d] | LC124921[d] | LC124955[d] |
| *Solenodon paradoxu* s, Hispaniolan solenodon | From Database | JN414026[e] | AY530069[h] | AY530070[h] | AY530080[h] | - |
| **Erinaceidae** | | | | | | |
| **Erinaceinae** | | | | | | |
| *Erinaceus amurensis* , Amur hedgehog | SDO12misc-2: Shizuoka, Japan | LC124833[d] | LC124860[d] | LC124889[d] | LC124922[d] | LC124956[d] |
| *Erinaceus amurensis* , Amur hedgehog | SDO12misc-3: Shizuoka, Japan | LC124834[d] | LC124861[d] | LC124890[d] | LC124923[d] | LC124957[d] |
| *Erinaceus europaeus* , West European hedgehog | From Database | JN414024[e] | - | - | AF284008[l] | - |
| *Erinaceus concolor* , southern white-breasted hedgehog | From Database | - | AY011383[i] | AY011447[i] | - | AY011871[i] |
| *Paraechinus aethiopicus* , desert hedgehog | QU1: northern Qatar | LC124835[d] | LC124862[d] | LC124891[d] | LC124924[d] | LC124958[d] |
| *Paraechinus aethiopicus* , desert hedgehog | QU2: northern Qatar | LC124836[d] | LC124863[d] | LC124892[d] | LC124925[d] | LC124959[d] |
| **Galericinae** | | | | | | |
| *Hylomys cf. suillus* , short-tailed gymnure | SDO-120701-1*: northern Vietnam | LC124837[d] | - | LC124893[d] | LC124926[d] | LC124960[d] |
| *Hylomys cf. suillus* , short-tailed gymnure | SDO-120702-2*: northern Vietnam | LC124838[d] | - | LC124894[d] | LC124927[d] | LC124961[d] |
| *Podogymnura truei* , Mindanao gymunure | From Database | JN414025[e] | JN633737[e] | JN633375[e] | - | - |
| **Soricidae** | | | | | | |
| **Crocidurinae** | | | | | | |
| *Crocidura dsinezumi* , dsinezumi shrew | SDO-11/02/23-2: Yakushima Is., Japan | - | LC124864[d] | LC124895[d] | LC124928[d] | LC124962[d] |
| *Suncus murinus* , Asian house shrew | SDO-11/12/7-1: Yonaguni Is., Japan | - | LC124865[d] | LC124896[d] | LC124929[d] | LC124963[d] |
| **Soricinae** | | | | | | |
| *Anourosorex yamashinai* , Taiwanese mole shrew | SDO-03/3/12-5: Chiayi Country, Taiwan | LC124839[d] | LC124866[d] | LC124897[d] | LC124930[d] | - |
| *Blarina brevicauda* , northern short-tailed shrew | SDO-2002/7/25-5: Michigan, USA | LC124840[d] | LC124867[d] | LC124898[d] | LC124931[d] | LC124964[d] |

| | | | | | | |
|---|---|---|---|---|---|---|
| *Blarinella griselda* , Indochinese short-tailed shrew | AMNH101610: Mt. Tay Con Linh, Vietnam | LC124841[d] | LC124868[d] | LC124899[d] | LC124932[d] | LC124965[d] |
| *Chimarrogale platycephalus* , Japanese water shrew | SDO-2001/5/25-1: Aomori Pref., Japan | LC124842[d] | LC124869[d] | LC124900[d] | LC124933[d] | - |
| *Chodsigoa sodalis* , lesser Taiwanese shrew | SDO-02misc74: Chiayi Country, Taiwan | - | LC124870[d] | LC124901[d] | LC124934[d] | - |
| *Cryptotis parva* , North American least shrew | ASNHC8192: Texas, USA | LC124843[d] | - | LC124902[d] | LC124935[d] | LC124966[d] |
| *Episoriculus fumidus* , Taiwanese brown-toothed shrew | SDO-03/3/11-1: Chiayi Country, Taiwan | - | LC124871[d] | LC124903[d] | LC124936[d] | - |
| *Neomys fodiens* , Eurasian water shrew | SDO95misc-20: Koverhar, S Finland | - | LC124872[d] | LC124904[d] | LC124937[d] | - |
| *Notiosorex crawfordi* , Crawford's gray shrew | ASNHC10653: Texas, USA | LC124844[d] | LC124873[d] | LC124905[d] | LC124938[d] | LC124967[d] |
| *Sorex araneus* , common shrew | From Database | JN414027[e] | AY011386[i] | AY011450[i] | AY057828[i] | - |
| *Sorex caecutiens* , Laxmann's shrew | SDO-100825-1: Hokkaido, Japan | LC124845[d] | LC124874[d] | LC124906[d] | LC124939[d] | LC124968[d] |
| **Talpidae** | | | | | | |
| **Scalopinae** | | | | | | |
| *Scalopus aquaticus* , eastern mole | From Database | - | - | - | AF284007[l] | - |
| **Talpinae** | | | | | | |
| *Scaptonyx fusicaudus* , long-tailed mole | From Database | - | - | - | - | AB106241[m] |
| *Euroscaptor klossi* , Kloss's mole | From Database | - | - | - | - | AB185155[n] |
| *Euroscaptor mizura* , Japanese mountain mole | SDO-2001/5/26-2: Aomori, Japan | LC124846[d] | LC124875[d] | LC124907[d] | LC124940[d] | LC124969[d] |
| *Mogera imaizumii* , small Japanese mole | From Database | - | - | - | - | AB106242[m] |
| *Mogera insularis* , insular mole | From Database | - | - | - | - | AB176544[m] |
| *Mogera tokudae* , Sado mole | From Database | - | - | - | - | AB106243[m] |
| *Mogera wogura* , Japanese mole | SDO-11/02/24-2: Yakushima Is., Japan | LC124847[d] | LC124876[d] | LC124908[d] | LC124941[d] | LC124970[d] |
| *Scaptochirus moschatus* , short-faced mole | From Database | - | - | - | - | AB353298[o] |
| *Talpa altaica* , Altai mole | From Database | JN414028[e] | HG738086[j] | AY011448[i] | - | AB176542[m] |
| *Talpa europaea* , European mole | From Database | AY243373[f] | - | - | - | - |
| *Dymecodon pilirostris* , True's shrew mole | SDO-96misc-8: Nagano, Japan | LC124848[d] | LC124877[d] | LC124909[d] | LC124942[d] | LC124971[d] |
| *Urotrichus talpoides* , Japanese shrew mole | SDO11misc7: Okayama, Japan | - | LC124878[d] | LC124910[d] | LC124943[d] | LC124972[d] |
| *Urotrichus talpoides* , Japanese shrew mole | FACT156: Fukuyama, Hiroshima, Japan | - | - | - | LC124944[d] | LC124973[d] |
| **Uropsilinae** | | | | | | |
| *Uropsilus gracilis* , gracile shrew mole | From Database | - | - | - | - | AB106240[m] |
| **Outgroups** | | | | | | |
| *Herpestes auropunctatus* , small Asian mongoose | SDO-12/8/23-2: Grande Comore Island | LC124849[d] | - | LC124911[d] | LC124945[d] | LC124974[d] |

| | | | | | | |
|---|---|---|---|---|---|---|
| *Mustela erminea* , ermine or stoat | From Database | AB193414[g] | EF987575[k] | EF987619[k] | AB285357[g] | AB109347[p] |
| *Mustela itatsi* , Japanese weasel | SDO-11misc5: Yakushima Is., Japan | LC124850[d] | LC124879[d] | LC124912[d] | LC124946[d] | LC124975[d] |
| *Mus musculus* , house mouse | SDO-11/02/24-4: Yakushima Is., Japan | LC124851[d] | LC124880[d] | LC124913[d] | LC124947[d] | LC124976[d] |
| *Tupaia belangeri* , northern treeshrew | SDO-13/01/16-1*: southern Vietnam | LC124852[d] | LC124881[d] | LC124914[d] | LC124948[d] | - |

[a]: Scientific and common names follow Hutterer (2005).

[b]: Voucher and locality information are provided for new data only (AMAR, collections in the project named Academic Mission of Almiqui Research; SDO, Satoshi D. Ohdachi's personal collections; QU, Qatar University; AMNH, American Museum of Natural History; ASNHC, Angelo State Natural History Collections; FACT, Fukuyama University Animal Cell Technology). *Samples are deposited at Institute of Eclogy and Biological Resources, Vietnam

[c]: Alejandro de Humboldt National Park

[d]: this study

[e]: Meredith, R. W. *et al* . Impacts of the Cretaceous Terrestrial Revolution and KPg extinction on mammal diversification. *Science* **334,** 521-524 (2011).

[f]: Amrine-Madsen, H., Koepfli, K.-P., Wayne, R. K. & Springer, M. S. A new phylogenetic marker, apolipoprotein B, provides compelling evidence for eutherian relationships. *Mol. Phylogenet. Evol.* **28,** 225-240 (2003).

[g]: Sato, J. J. *et al* . Deciphering and dating the red panda’s ancestry and early adaptive radiation of Musteloidea. *Mol. Phylogenet. Evol.* **53,** 907-922 (2009).

[h]: Roca, A. L. *et al* . Mesozoic origin for west indian insectivores. *Nature* **429,** 649-651 (2004).

[i]: Murphy,W.J. *et al* . Molecular phylogenetics and the origins of placental mammals. *Nature* **409,** 614-618 (2001).

[j]: He, K., Shinohara, A., Jiang, X.-L. & Campbell, K. L. Multilocus phylogeny of talpine moles (Talpini, Talpidae, Eulipotyphla) and its implications for systematics. *Mol. Phylogenet. Evol.* **70,** 513-521 (2014).

[k]: Koepfli, K.-P. *et al* . Multigene phylogeny of the Mustelidae: resolving relationships, tempo and biogeographic history of a mammalian adaptive radiation. *BMC Biology* **6,** 10 (2008).

[l]: Madsen, O. *et al* . Parallel adaptive radiations in two major clades of placental mammals. *Nature* **409,** 610-614 (2001).

[m]: Shinohara, A. *et al* . Evolution and Biogeography of Talpid Moles from Continental East Asia and the Japanese Islands Inferred from Mitochondrial and Nuclear Gene Sequences. *Zoolog. Sci.* **21,** 1177-1185 (2004).

[n]: Kawada, S., Shinohara, A., Yasuda, M., Oda, S. & Liat, L. B. The mole of Peninsular Malaysia: notes on its identification and ecology. *Mamm. Study* **28,** 73-77 (2003).

[o]: Shinohara, A. *et al* . Phylogenetic relationships of the short-faced mole, Scaptochirus moschatus (Mammalia: Eulipotyphla), among Eurasian fossorial moles, as inferred from mitochondrial and nuclear gene sequences. *Mamm. Study* **33,** 77-82 (2008).

Supplementary Table S2. Time to the most recent common ancester (TMRCA) and 95 Credibility interval of each clade in the eulipotyphlan phylogeny (Supplementary Fig. 1)

| clade | TMRCA (MYA) | | |
|---|---|---|---|
| | strategy [1] | strategy [2] | strategy [3] |
| 1_Eulipotyphla | 58.6 (57.3-60.8) | 75.7 (74.5-76.9) | 75.9 (74.7-77.1) |
| 2_Solenodontidae | 3.7 (2.6-5.0) | 4.8 (3.3-6.4) | 4.6 (3.1-6.0) |
| 3_Talpidae-Soricidae-Erinaceidae | 58.6 (57.3-60.8) | 74.2 (64.4-77.4)*(64) | 75.9 (74.7-77.1) |
| 4_Talpidae | 35.4 (34.2-38.5)*(137) | 36.2 (34.2-43.2)*(121) | 29.3 (20.3-42.9)*(114) |
| 5_Talpinae-Scalopinae | 35.0 (34.3-35.9) | 35.1 (34.3-36.1) | 23.7 (19.3-28.6)*(193) |
| 6_Talpinae | 19.8 (16.1-23.5) | 25.2 (20.9-29.2) | 21.7 (17.9-25.1) |
| 7_*Scaptonyx* -*Dimecodon* -*Urotrichus* | 14.3 (8.0-20.5)*(188) | 18.2 (9.9-26.2)*(183) | 17.8 (10.0-24.7)*(147) |
| 8_*Dimecodon* -*Urotrichus* | 2.3 (1.3-3.5) | 2.9 (1.5-4.4) | 2.7 (1.6-4.1) |
| 9_*Talpa* -*Mogera* -*Euroscaotor* -*Scaptochirus* | 7.1 (5.2-9.2) | 9.0 (6.7-11.8) | 8.5 (6.2-11.0) |
| 10_*Mogera* -*Euroscaotor* -*Scaptochirus* | 3.7 (2.5-5.1) | 4.8 (3.2-6.5) | 4.5 (3.0-6.1) |
| 11_*Euroscaotor* -*Scaptochirus* | 2.9 (1.2-4.8) | 3.7 (1.3-5.9) | 3.4 (1.3-5.7)*(192) |
| 12_*E. malayana* -*S. moschatus* | 1.7 (0.4-3.2) | 2.2 (0.4-4.1) | 2.1 (0.5-3.8) |
| 13_*Mogera* | 2.0 (0.9-3.3) | 2.6 (1.0-4.3) | 2.3 (0.8-4.0) |
| 14_*M. imaizumi* -*M. tokudae* -*M. wogura* | 1.4 (0.3-2.6) | 1.8 (0.5-3.4) | 1.7 (0.3-3.1) |
| 15_*M. imaizumi* -*M. tokudae* | 0.8 (0.1-1.7) | 1.0 (0.1-2.3) | 1.0 (0.1-2.1) |
| 16_Erinaceidae-Soricidae | 58.5 (57.3-60.4) | 73.0 (59.6-77.3)*(63) | 75.9 (74.7-77.1) |
| 17_Erinaceidae | 39.6 (38.1-41.5) | 41.4 (38.4-45.0) | 40.3 (35.0-46.0) |
| 18_Galericinae | 18.7 (13.6-24.0) | 21.3 (15.9-26.5) | 20.6 (15.1-26.4) |
| 19_Erinaceinae | 3.9 (2.9-5.1) | 4.9 (3.6-6.4) | 4.7 (3.3-6.1) |
| 20_*Erinaceus* | 1.1 (0.6-1.8) | 1.4 (0.7-2.1) | 1.3 (0.7-2.0) |
| 21_*E. amurensis* -*E. europaeus* | 1.0 (0.6-1.6) | 1.3 (0.7-1.9) | 1.2 (0.7-1.8) |
| 22_Soricidae | 34.3 (29.9-38.5) | 44.1 (36.8-50.4)*(105) | 48.0 (42.3-53.8) |
| 23_Crocidurinae | 6.0 (4.0-8.0) | 7.8 (5.2-10.3) | 7.9 (5.6-10.4) |
| 24_Soricinae | 26.5 (23.5-29.7)*(124) | 34.0 (29.6-38.1)*(99) | 35.0 (31.3-38.7) |
| 25_*B. brevicauda* -*C. parva* -*B. griselda* -*Sorex* | 22.5 (19.6-25.5)*(152) | 28.9 (24.8-32.9)*(111) | 30.0 (26.3-33.5) |
| 26_*Sorex* | 3.4 (2.2-4.9) | 4.3 (2.8-6.0) | 4.2 (2.7-5.8) |
| 27_*B. brevicauda* -*C. parva* -*B. griselda* | 12.8 (10.4-15.4) | 16.4 (13.4-19.5) | 16.6 (13.6-19.5) |
| 28_*B. brevicauda* -*C. parva* | 5.8 (4.2-7.5) | 7.3 (5.3-9.4) | 7.3 (5.4-9.3) |
| 29_*C. sodalis* -*E. fumidus* -*C. platycephalus* -*N. fodiens* -*N. crawfordi*-*A. yamashinai* | 21.3 (18.5-24.4)*(165) | 27.3 (23.4-31.2)*(141) | 27.9 (24.2-31.8) |
| 30_*C. sodalis* -*E. fumidus* -*C. platycephalus* -*N. fodiens* -*N. crawfordi* | 14.6 (12.2-17.3)*(186) | 18.4 (15.4-21.5) | 18.5 (15.4-21.5) |
| 31_*C. sodalis* -*E. fumidus* -*C. platycephalus* -*N. fodiens* | 8.5 (6.7-10.5)*(153) | 10.6 (8.4-12.9)*(163) | 10.4 (8.2-12.7)*(175) |
| 32_*C. sodalis* -*E. fumidus* -*C. platycephalus* | 8.3 (6.5-10.3)*(160) | 10.3 (8.1-12.7)*(172) | 10.1 (7.9-12.3)*(187) |
| 33_*C. sodalis* -*E. fumidus* | 8.0 (6.0-10.0)*(188) | 10.0 (7.5-12.5) | 9.8 (7.4-12.3) |

*: ESS < 200 and each ESS is shown in the parenthesis after each astarisk

Supplementary Table S3. Primers adopted in the PCR and DNA sequencing.

| Gene | Primers | | | | |
|---|---|---|---|---|---|
| | Name | 1st/2nd PCR | Forward/Reverse | Sequence (5' to 3') | References |
| *Apob* | 187F | 1st and 2nd | Forward | GTG CCA GGT TCA ATC AGT ATA AGT | Amrine-Madsen et al. (2003) |
| | APOB-F9214-Eri | 2nd | Forward | CCA GCT CTG GGA AAC ATT ACC TG | this study |
| | APOB-F9287 | 2nd | Forward | TAT AAC CAG TCA GAT ATT GTT GCT | Sato et al. (2006) |
| | APOB-F8830-Sori | 2nd | Forward | CAG GCA AGT TGA GAA AGT CAG | this study |
| | APOB-R8922-Sori | 2nd | Reverse | GCC TGG AAT TTG AAA GGT CCT TGG | this study |
| | APOB-R9324 | 2nd | Reverse | GGT GCC CTC TAA TTT GTA CTG CAG | Sato et al. (2006) |
| | APOB-R9600-Eri | 2nd | Reverse | CCT GTG GCA GTG GTG TAC ACT CT | this study |
| | J1R | 1st and 2nd | Reverse | CCA GCA AAA TTT TCT TTT ACT TCA A | Jiang et al. (1998) |
| *Atp7a* | ATP7A-Fw | 1st | Forward | TCC CTG GAC AAT CAA GAA GC | Murphy et al. (2001) |
| | ATP7A-Rv | 1st | Reverse | AAG GTA GCA TCA AAT CCC ATG T | Murphy et al. (2001) |
| *Bdnf* | BDNF-Fw | 1st | Forward | CAT CCT TTT CCT TAC TAT GGT T | Murphy et al. (2001) |
| | BDNF-Rv | 1st | Reverse | TTC CAG TGC CTT TTG TCT ATG | Murphy et al. (2001) |
| *Brca1* | BRCA1-F997 | 1st and 2nd | Forward | GAG AAC AGC AGT TTA TTA CTC AC | Sato et al. (2009) |
| | BRCA1-F1428 | 2nd | Forward | AGA CTT AAT GGC CAG TGA TCC TC | Sato et al. (2009) |
| | BRCA1-R1509 | 2nd | Reverse | AGG CTT GCC TTC CTC CGA TAG GT | Sato et al. (2009) |
| | BRCA1-R2047 | 1st and 2nd | Reverse | CAT CTC TTC ACT GCT AGA ACA AC | Sato et al. (2009) |
| *Rag1* | RAG1-F1842 | 1st and 2nd | Forward | GCT TTG ATG GAC ATG GAA GAA GAC AT | Teeling et al. (2000, their RAG1F1705) |
| | RAG1-F2357 | 2nd | Forward | AGC CTC CCA AAA TCT TGT CTT CCA CTC CA | Sato et al. (2004) |
| | RAG1-F2357-Cro | 2nd | Forward | GGCCTCGCAGAATCTGGTCTTCCATTCCA | this study |
| | RAG1-R2388-CroEri | 2nd | Reverse | GGG TTG GAA CGC CAG ACC TCA TAG C | this study |
| | RAG1-R2486 | 2nd | Reverse | AAT GTC ACA GTG AAG GGC ATC TAT GGA AGG | Sato et al. (2004) |
| | RAG1-R2951 | 1st and 2nd | Reverse | GAG CCA TCC CTC TCA ATA ATT TCA GG | Teeling et al. (2000, their RAG1R2864) |
